# High-resolution three‑dimensional contrast‑enhanced magnetic resonance venography in children: comparison of gadofosveset trisodium with ferumoxytol

**DOI:** 10.1007/s00247-021-05225-2

**Published:** 2021-12-22

**Authors:** Puja Shahrouki, Sarah N. Khan, Takegawa Yoshida, Paul J. Iskander, Shahnaz Ghahremani, J. Paul Finn

**Affiliations:** 1 Diagnostic Cardiovascular Imaging Research Laboratory, Department of Radiological Sciences, University of California at Los Angeles, Peter V. Ueberroth Building, Suite 3371, 10945 Le Conte Ave, Los Angeles, CA 90095-7206 USA; 2 Division of Pediatric Radiology, Department of Radiological Sciences, University of California at Los Angeles, Los Angeles, CA USA; 3 Department of Radiology, Children’s Hospital Los Angeles, Los Angeles, CA USA

**Keywords:** Children, Congenital heart disease, Ferumoxytol, Gadofosveset, Magnetic resonance imaging, Magnetic resonance venography

## Abstract

**Background:**

Gadofosveset is a gadolinium-based blood pool contrast agent that was approved by the United States Food and Drug Administration in 2008. Its unanticipated withdrawal from production in 2016 created a void in the blood pool agent inventory and highlighted the need for an alternative agent with comparable imaging properties.

**Objective:**

The purpose of our study is to compare the diagnostic image quality, vascular contrast-to-noise ratio (CNR) and temporal signal characteristics of gadofosveset trisodium and ferumoxytol at similar molar doses for high-resolution, three-dimensional (3-D) magnetic resonance (MR) venography in children.

**Materials and methods:**

The medical records and imaging data sets of patients who underwent high-resolution 3-D gadofosveset-enhanced MR venography (GE-MRV) or ferumoxytol-enhanced MR venography (FE-MRV) were retrospectively reviewed. Two groups of 20 pediatric patients (age- and weight-matched with one patient common to both groups; age range: 2 days–15 years) who underwent high-resolution 3-D GE-MRV or FE-MRV at similar molar doses were identified and analyzed. Qualitative analysis of image quality and vessel definition was performed by two blinded pediatric radiologists. Interobserver agreement was assessed with the AC1 (first-order agreement coefficient) statistic. Signal-to-noise ratio (SNR) and CNR of the inferior vena cava and aorta were measured in the steady-state venous phase. Medical records were retrospectively reviewed for any adverse reactions associated with either contrast agent.

**Results:**

Measured SNR and CNR of the inferior vena cava were higher for FE-MRV than GE-MRV (*P* = 0.034 and *P* < 0.001, respectively). The overall image quality score and individual vessel scores of FE-MRV were equal to or greater than GE-MRV (*P* = 0.084), with good interobserver agreement (AC1 = 0.657). The venous signal on FE-MRV was stable over the longest interval measured (1 h, 13 min and 46 s), whereas venous signal on GE-MRV showed more variability and earlier loss of signal. No adverse reactions were noted in any patient with either contrast agent.

**Conclusion:**

Ferumoxytol produces more uniform and stable enhancement throughout the entire venous circulation in children than gadofosveset, offering a wider time window for optimal image acquisition. FE-MRV offers a near-ideal approach to high-resolution venography in children at all levels of anatomical complexity.

**Supplementary Information:**

The online version contains supplementary material available at 10.1007/s00247-021-05225-2.

## Introduction

Venous imaging in pediatric patients is performed for a variety of clinical indications, including pre- and post-organ transplant evaluation, locating access sites for venous cannulation and pre- or postsurgical assessment in congenital heart disease. Computed tomography (CT) has drawbacks in this population due to challenges with contrast bolus timing and concerns about ionizing radiation and contrast nephrotoxicity [1, 2]. Although vascular ultrasound (US) remains a first-line test, the lack of suitable acoustic windows limits its role in several anatomical regions [3]. For these reasons, magnetic resonance imaging (MRI) has assumed an increasingly important role in pediatric vascular imaging.

Gadofosveset trisodium (Ablavar; Lantheus Medical Imaging, North Billerica, MA) is a gadolinium-based blood pool contrast agent approved by the United States Food and Drug Administration (FDA) in 2008 for the evaluation of aortoiliac disease in adults [4], but it was withdrawn from production in 2016 for commercial reasons. Relative to the extracellular gadolinium-based contrast agents (GBCAs), reversible binding to serum albumin endowed gadofosveset with a high R1 relaxivity (19 s^−1^ mM^−1^ at 1.5 T [T] and 9.9 s^−1^ mM^−1^ at 3.0 T) [5], and a prolonged initial plasma distribution half-life (on the order of 30 min) before undergoing renal (91%) and biliary (9%) excretion [6, 7]. Gadofosveset had become favored in pediatric practice for the classification of vascular and soft-tissue anomalies of the head and neck [8], and for cardiovascular imaging in the chest and abdomen [9]. Among the GBCAs, gadofosveset was the de facto reference standard for venographic imaging in children. However, its unanticipated withdrawal from production created a void in the blood pool agent inventory and highlighted the need for an alternative agent with comparable imaging properties.

Ferumoxytol (Feraheme; AMAG Pharmaceuticals, Waltham, MA) is an ultra-small superparamagnetic iron oxide (USPIO) nanoparticle, approved and marketed in the U.S. for parenteral treatment of iron deficiency. It was originally designed as a blood pool agent for contrast-enhanced MR angiography [10, 11], and recently has generated interest as an alternative to the GBCAs for a variety of MR imaging applications [12]. Ferumoxytol has a mean particle diameter of 30 nm, an intravascular half-life of ~ 14 h and high R1 relaxivity at both 1.5 T (19 s^−1^ mM^−1^) and at 3.0 T (10 s^−1^ mM^−1^) [13], which are desirable properties for steady-state bright blood imaging.

Although ferumoxytol has been used successfully for vascular MRI in children [14–17], no direct comparisons of ferumoxytol to gadofosveset have been reported in this patient group. As interest grows in the off-label diagnostic use of ferumoxytol, it is relevant to assess its performance relative to the prior reference intravascular agent, gadofosveset.

The purpose of our study, therefore, is to compare the diagnostic image quality and vascular contrast-to-noise ratio (CNR) over time using gadofosveset and ferumoxytol for high-resolution MR venography in children.

## Materials and methods

This study was approved by the local institutional review board and was compliant with the Health Insurance Portability and Accountability Act. The medical records and imaging data sets of patients who underwent high-resolution three-dimensional (3-D) gadofosveset-enhanced MR venography (GE-MRV) or ferumoxytol-enhanced MR venography (FE-MRV) were retrospectively reviewed.

### Study population

Two groups of 20 pediatric patients (age- and weight-matched with one patient common to both groups; age range: 2 days–15 years) who underwent high-resolution 3-D GE-MRV or FE-MRV at similar molar doses were identified and analyzed.

Table 1 summarizes the demographic details for all 39 patients included in the study. The clinical indications for the contrast-enhanced studies were varied, but a majority of those studied with ferumoxytol (*n* = 13, 65%) had congenital heart disease. Of the patients studied with gadofosveset, 45% (*n* = 9) had congenital heart disease. Since MRI studies in children with congenital heart disease are typically time-consuming, images acquired later in the examination (e.g., following cine cardiac imaging and flow measurement) afford the opportunity to sample the MR signal evolution over a longer period than would be the case for less complex vascular studies that involve running fewer pulse sequences. For the patients undergoing FE-MRV and GE-MRV, the female-to-male distribution was equal, the mean age was 4.2 ± 4.9 years and 4.1 ± 5.1 years, respectively (*P* = 0.92), and the mean weight was 15.5 ± 14.3 kg and 13.6 ± 13.0 kg, respectively (*P* = 0.66). One patient underwent GE-MRV and FE-MRV. This patient was common to both groups and is the only patient in the cohort who was not age- and weight-matched for the inter-agent comparison. Patients with implantable intracardiac devices were excluded.

**Table 1:** Patient demographics

	GE-MRV	FE-MRV	*P*-value
Total in study (*n*)	20	20	
Females studied (*n*)	10	10	
Age range (years)	0–15.5	0–15.2	
Mean age ± standard deviation (years)	4.1 ± 5.1	4.2 ± 4.9	0.92
Weight range (kg)	1.0–40.8	1.6–51.4	
Mean weight ± standard deviation (kg)	13.6 ± 13.0	15.5 ± 14.3	0.66

*FE-MRV* ferumoxytol-enhanced magnetic resonance venography, *GE-MRV* gadofosveset-enhanced magnetic resonance venography

### Image acquisition

Retrospective analysis of GE-MRV and FE-MRV data sets revealed that younger patients and those unable to follow breath-hold instructions were examined under general anesthesia with controlled ventilation (gadofosveset [*n* = 17; 85%], ferumoxytol [*n* = 17; 85%]). Older patients who could cooperate with breath-hold instructions were examined without sedation (gadofosveset [*n* = 3; 15%], ferumoxytol [*n* = 2; 10%]). General anesthesia was administered by pediatric anesthesiologists [14]. Muscle relaxation was established with a non-depolarizing neuromuscular blocker (rocuronium bromide) and anesthesia was maintained with a fluorinated inhalational agent (isofluorane, 1.0–1.5%). Positive pressure ventilation was performed with an MR imaging-compatible ventilator (Narkomed MRI_2; Drager Medical, Telford, PA). During breath-held image acquisition, the ventilator was paused.

MR imaging was performed on a 3.0 T Magnetom TIM Trio imaging system (Siemens Medical Solution, Malvern, PA) (Table 2). Depending on patient size, a combination of head–neck, spine and phased-array multi-element surface coils were used for signal reception. Multiplanar survey images were initially obtained using half-Fourier acquisition single-shot turbo spin echo (HASTE), followed by high spatial resolution contrast-enhanced MR angiography with a spoiled 3-D gradient echo sequence. There were no relevant changes to the MRI hardware or software specifications throughout the time period encompassing the patient studies reported here. Contrast-enhanced MR venography images were acquired in the venous phase, more than 1 min post injection, when the contrast agent had time to distribute fully through the vascular system. Total gadofosveset dosage was 0.06 mmol/kg, administered by bolus injection. Stock ferumoxytol was diluted with normal saline by a factor of 10 and administered to a total dose of 4 mg/kg (equivalent to 0.07 mmol/kg). The FE-MRV studies were performed before March 2015, at which time the FDA issued a boxed warning against fast bolus injection and recommended that ferumoxytol be administered by slow infusion over several minutes [15].

**Table 2:** Example technical parameters for contrast-enhanced MR angiography acquisitions^a^

	GE-MRV	FE-MRV
TR (ms)	3.38	3.02
TE (ms)	1.35	1.16
Flip angle	12°	15°
Bandwidth (Hertz/pixel)	610	610
Voxel dimensions (mm^3^)	0.6–1.2 × 0.6–1.2 × 0.6–1.2 = 0.2–1.7	0.6–1.2 × 0.6–1.2 × 0.6–1.2 = 0.2–1.7
Number of phase encoding steps	162	149
Acquisition time (s)	16.4–21	16.0–21
Specific absorption rate	0.434	0.627
Parallel acquisition factor	3	3

*FE-MRV* ferumoxytol-enhanced magnetic resonance venography, *GE-MRV* gadofosveset-enhanced magnetic resonance venography, *TE* echo time, *TR* repetition time

^a^ 3.0 T, 32-channel whole-body scanner (Magnetom TIM Trio), gradient strength = 40 mT/m, slew rate = 200 mT/m/ms

### Qualitative image analysis

For both agents, two independent pediatric radiologists (P.J.I. and S.G.), each with more than 5 years of post-fellowship experience in cardiovascular imaging, who were blinded to the contrast material used and all clinical data, performed qualitative assessments, in a randomized order, of the partition images and thin maximum intensity projection (MIP) images in the venous phase.

The veins were divided into 18 segments: internal jugular veins, subclavian veins, innominate veins, superior vena cava (SVC), superior pulmonary veins, inferior pulmonary veins, tributaries of the superior and inferior pulmonary veins, inferior vena cava (IVC), azygos vein, common iliac veins, external iliac veins, internal iliac veins, superior mesenteric vein, tributaries of the superior mesenteric vein, inferior mesenteric vein, middle hepatic vein, splenic vein, and portal vein. Vascular image quality was evaluated on a 4-point scale as previously described [16] and detailed in Online Supplementary Material 1. In addition, the images were assessed for overall image quality on a 4-point scale (Online Supplementary Material 1).

### Quantitative image analysis

Signal intensity and standard deviations were measured within and adjacent to the inferior vena cava and aorta in the steady-state venous phase by a reviewer with 9 years of postgraduate experience (T.Y.). For noise measurement, the signal intensity and standard deviation were averaged from six locations outside the body. These locations were matched as closely as possible in both groups. Signal-to-noise ratio (SNR) was calculated as the signal intensity of the segment of interest divided by the standard deviation of the background noise. CNR was calculated as the difference in signal intensity between the segment of interest and the adjacent tissue divided by the standard deviation of the background noise [17].

### Statistical analysis

Continuous data are presented as means and standard deviations, and categorical data are presented as absolute values with frequencies. Normality was tested with the Shapiro–Wilk test. Qualitative and quantitative differences were compared with paired two-tailed *t*-tests or Wilcoxon rank sum tests as appropriate. Interobserver agreement was assessed with the AC1 (first-order agreement coefficient) statistic in place of Cohen’s kappa value because “kappa’s paradox” was present [18]. AC1 values of < 0.20, 0.21–0.40, 0.41–0.60, 0.61–0.80 and 0.81–1.00 correspond to poor, fair, moderate, good and excellent agreement, respectively. Statistical analysis was performed with SPSS software (version 26.0; IBM, Armonk, NY).

## Results

### Patient characteristics

Table 1 summarizes demographic details for all 39 patients included in the study. There were no adverse reactions to either gadofosveset or ferumoxytol and vital signs remained stable throughout the procedures. The average time for the latest steady-state acquisition was 26 min and 54 s (range: 1 min and 38 s to 1 h, 13 min and 46 s) for FE-MRV and 5 min and 2 s (range: 35 s to 21 min and 35 s) for GE-MRV. Examples of cardiovascular diagnoses made on the contrast-enhanced MRI studies are provided in Online Supplementary Material 2.

### Qualitative assessment

The vessel definition score was significantly higher for ferumoxytol in 16/18 segments (*P* < 0.05; Table 3). In the internal iliac and external iliac veins, the scores were not significantly different between gadofosveset and ferumoxytol (*P* > 0.05). Overall, subjective image quality scores were higher for ferumoxytol compared to gadofosveset (2.85 ± 0.56 and 2.38 ± 0.84, respectively; *P* = 0.084), but the differences were not statistically significant.

**Table 3:** Vessel definition score

	GE-MRV		FE-MRV		Paired *t-*test *P*-value
	Mean observer score ± SD	Interobserver agreement AC1 statistic	Mean observer score ± SD	Interobserver agreement AC1 statistic	
Inferior vena cava	2.53 ± 1.03	0.538	3.68 ± 0.44	0.824	< 0.001
Superior vena cava	2.53 ± 0.97	0.671	3.25 ± 0.75	0.751	0.020
Innominate vein	2.45 ± 1.09	0.867	3.35 ± 0.63	0.756	0.003
Internal jugular vein	2.73 ± 1.08	0.537	3.60 ± 0.53	0.642	0.003
Subclavian vein	2.50 ± 1.09	0.601	3.35 ± 0.67	0.753	0.006
Superior pulmonary vein	2.25 ± 0.85	0.545	2.83 ± 0.44	0.576	0.017
Inferior pulmonary vein	2.13 ± 0.83	0.415	2.80 ± 0.66	0.435	0.007
Tributaries of the superior and inferior pulmonary veins	2.05 ± 0.78	0.615	2.55 ± 0.46	0.634	0.008
Azygos vein	2.08 ± 0.96	0.476	2.95 ± 0.74	0.485	0.009
Superior mesenteric vein	2.40 ± 0.91	0.674	3.15 ± 0.88	0.679	0.014
Inferior mesenteric vein	2.00 ± 0.90	0.479	2.60 ± 0.81	0.409	0.005
Tributaries of the superior mesenteric vein	2.00 ± 0.87	0.545	2.68 ± 0.78	0.415	0.030
Splenic vein	2.05 ± 0.89	0.675	2.93 ± 0.61	0.489	0.001
Middle hepatic vein	2.18 ± 0.88	0.280	3.13 ± 0.54	0.437	< 0.001
Portal vein	2.50 ± 0.90	0.609	3.48 ± 0.57	0.817	< 0.001
Common iliac vein	2.45 ± 1.01	0.602	3.20 ± 1.15	0.754	0.038
Internal iliac vein	2.05 ± 1.03	0.479	2.58 ± 1.00	0.341	0.15
External iliac vein	2.28 ± 1.20	0.672	2.88 ± 1.25	0.549	0.17

*AC1* first order agreement coefficient, *FE-MRV* ferumoxytol-enhanced magnetic resonance venography, *GE-MRV* gadofosveset-enhanced magnetic resonance venography, *SD* standard deviation

The interobserver agreement for overall image quality was good (AC1 = 0.657). The interobserver agreement scores for the individual vessel segments varied from fair to excellent (AC1 = 0.280–0.867; Table 3).

**Fig. 1:**
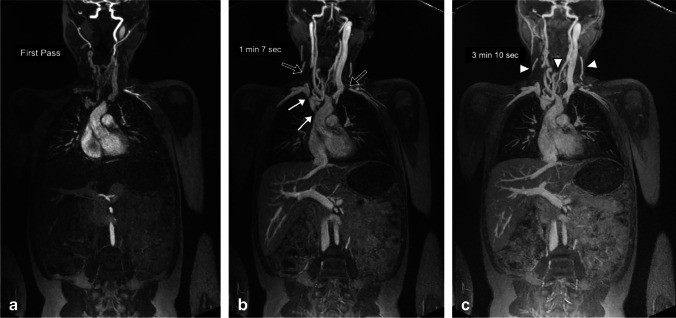
A 14-year-old girl with familial malabsorption syndrome requiring total parenteral nutrition. **a–c** Coronal gadofosveset-enhanced magnetic resonance angiography was performed at 3.0 T in the arterial (**a**), early venous (**b**) and late venous (**c**) phases to assess sites for central venous catheter placement. Thin maximum intensity projection images demonstrate occlusion of right internal jugular and left subclavian veins (*black arrows*), and stenosis of the right innominate vein and superior vena cava (*white arrows*) with anterior neck venous collaterals (*arrowheads*). Note the persistently good venous enhancement in the late venous phase

**Fig. 2:**
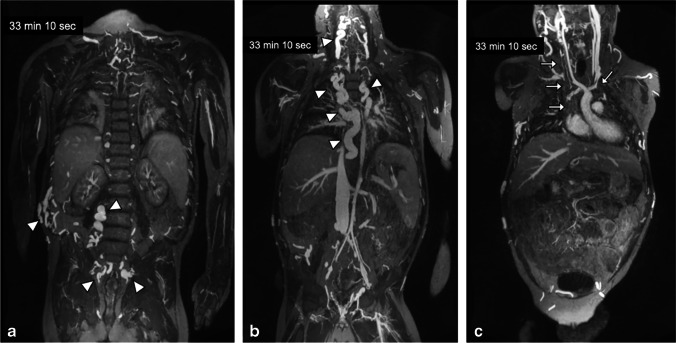
A 12-year-old boy with chronic renal failure requiring venous access. **a–c** Coronal ferumoxytol-enhanced magnetic resonance venography at 3.0 T in steady-state (33 min after injection) shows occlusion of the bilateral subclavian veins, innominate veins, right internal jugular vein and superior vena cava (*arrows*). Select thin maximum intensity projection reconstructions are shown. Note the uniformly high vascular signal throughout the venous system. Note the extensive venous collateralization in the neck, chest, abdomen and pelvis (*arrowheads*)

**Fig. 3:**
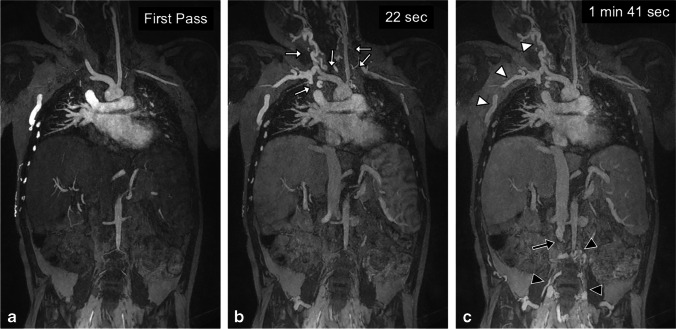
A 10-year-old boy post liver transplantation with extensive venous thrombosis. **a–c** Coronal gadofosveset-enhanced magnetic resonance angiography was performed in the arterial (**a**), early venous (**b**) and late venous (**c**) phases at 3.0 T to assess sites for central venous catheter placement. There is occlusion of the bilateral internal jugular veins, subclavian veins, innominate veins and distal superior vena cava (*white arrows*) with extensive chest wall and anterior neck collaterals (*white arrowheads*). The infrarenal inferior vena cava is occluded (*black arrow*) with dilated paravertebral collaterals (*black arrowheads*). Note the persistently good enhancement of the proximal inferior vena cava in early (22 s) and late (1 min, 41 s) venous phases

**Fig. 4:**
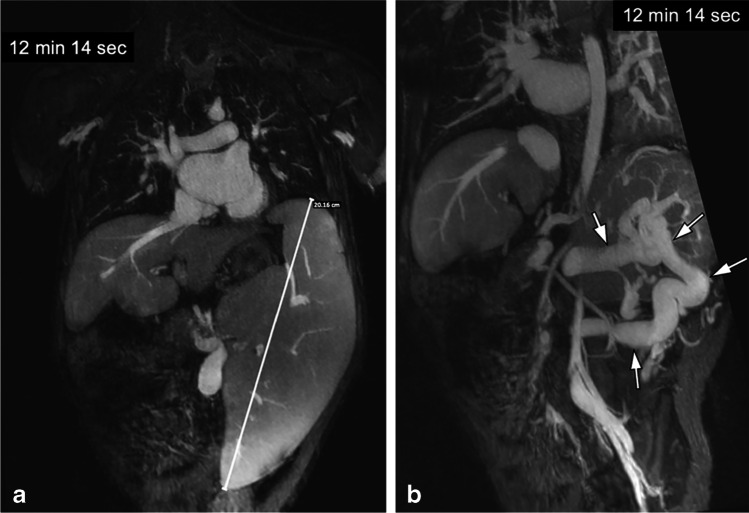
A 10-year-old girl with portal hypertension and splenomegaly. **a, b** Coronal ferumoxytol-enhanced magnetic resonance venography was performed at 3.0 T. The patient has gross splenomegaly shown on thin maximum intensity projection (MIP) (**a**) and a large spontaneous splenorenal shunt (*arrows*) shown on thick MIP (**b**). Note the uniform and high intravascular signal at 12 min post contrast injection

**Fig. 5:**
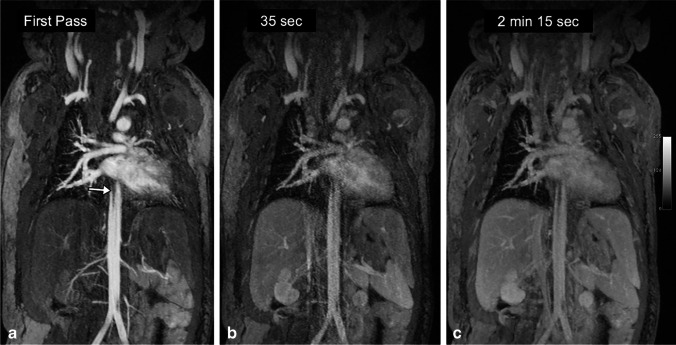
A 2-day-old boy with a history of truncus arteriosus. **a–c** Coronal thin maximum intensity projection images from gadofosveset-enhanced magnetic resonance angiography in the arterial (**a**), early venous (**b**) and late venous (**c**) phases at 3.0 T. The first pass arterial signal is high, but the venous signal is markedly lower in both early and late venous phases. The linear structure in the aorta is an umbilical artery catheter (*arrow*)

**Fig. 6:**
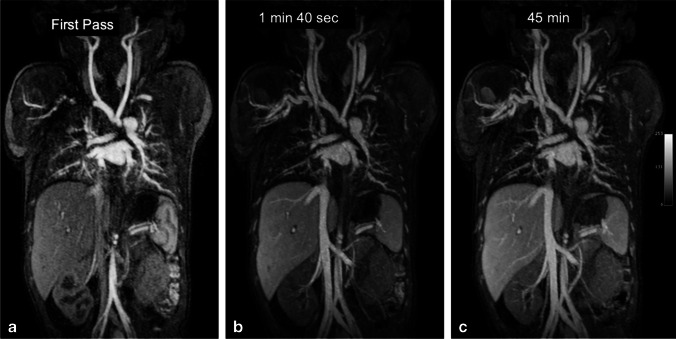
A 3-day-old boy with hypoplastic aortic arch and coarctation. **a–c** Coronal thin maximum intensity projection images from ferumoxytol-enhanced magnetic resonance angiography in the arterial (**a**), early venous (**b**) and late venous (**c**) phases at 3.0 T. Note the stable intravascular signal in the 43-min interval between the early and late venous phases

**Fig. 7:**
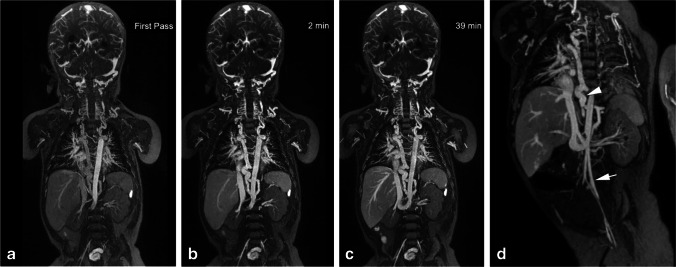
An 8-month-old boy post Norwood procedure and Glenn shunt for hypoplastic left heart syndrome, with abnormal venous anatomy. **a–c** Coronal thin maximum intensity projection (MIP) images from ferumoxytol-enhanced magnetic resonance angiography in the arterial (**a**), early venous (**b**) and late venous (**c**) phases at 3.0 T. Note the stable intravascular signal in the 37-min interval between the early and late venous phases. **d** Coronal thin MIP image shows an infrarenal left interior vena cava (IVC) (*arrow*) that crosses the midline to join the infrahepatic IVC. There is extensive venous collateralization (*arrowhead*) due to occlusion of the right superior vena cava, bilateral subclavian veins and right internal jugular vein

**Fig. 8:**
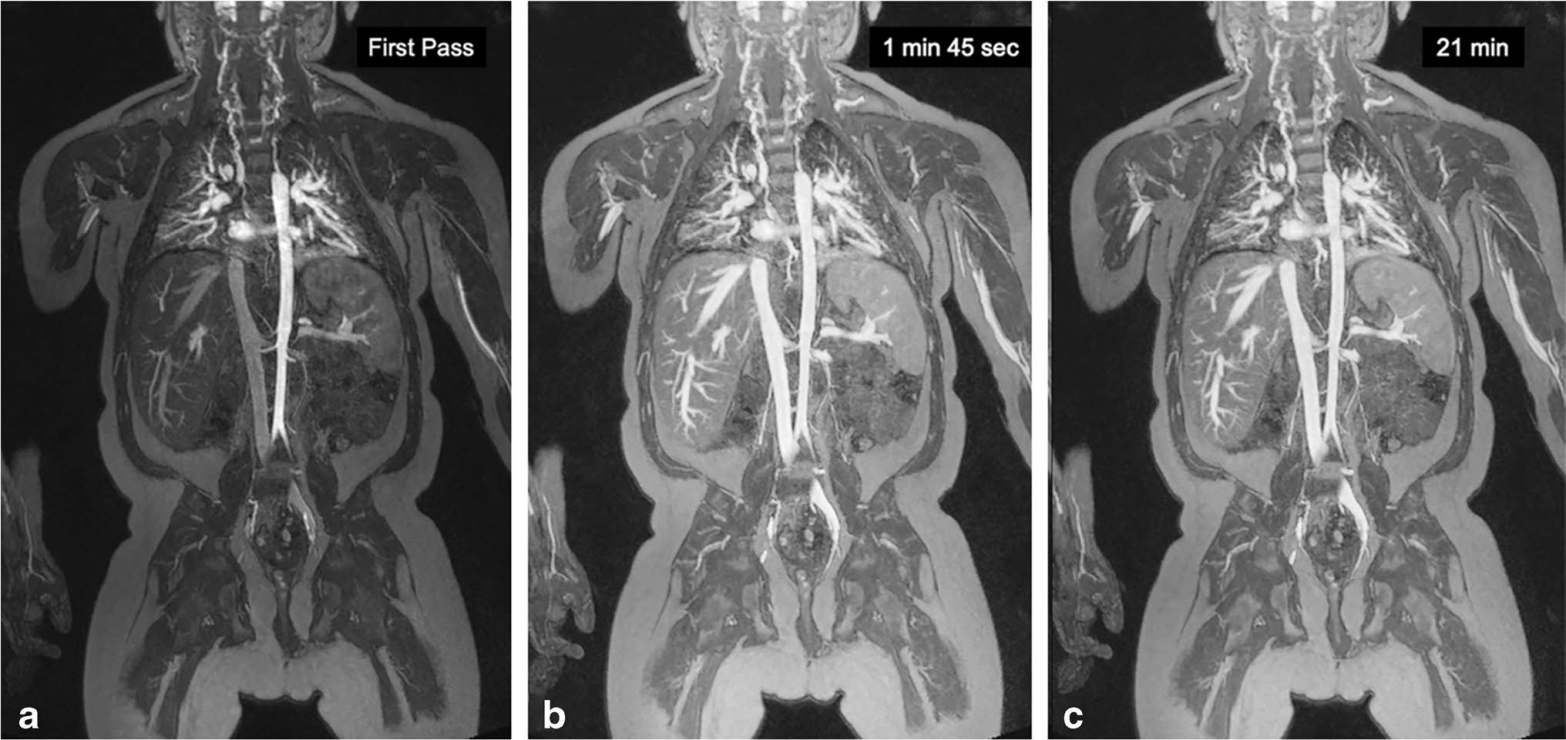
A 4-year-old boy with end-stage renal disease on hemodialysis. **a–c** Coronal ferumoxytol-enhanced magnetic resonance angiography was performed at 3.0 T to evaluate access sites for central venous catheter placement. Thin maximum intensity projection images are shown in the arterial (**a**), early venous (**b**) and late venous (**c**) phases. Note the stable intravascular signal in the 19-min interval between the early and late venous phases

**Fig. 9:**
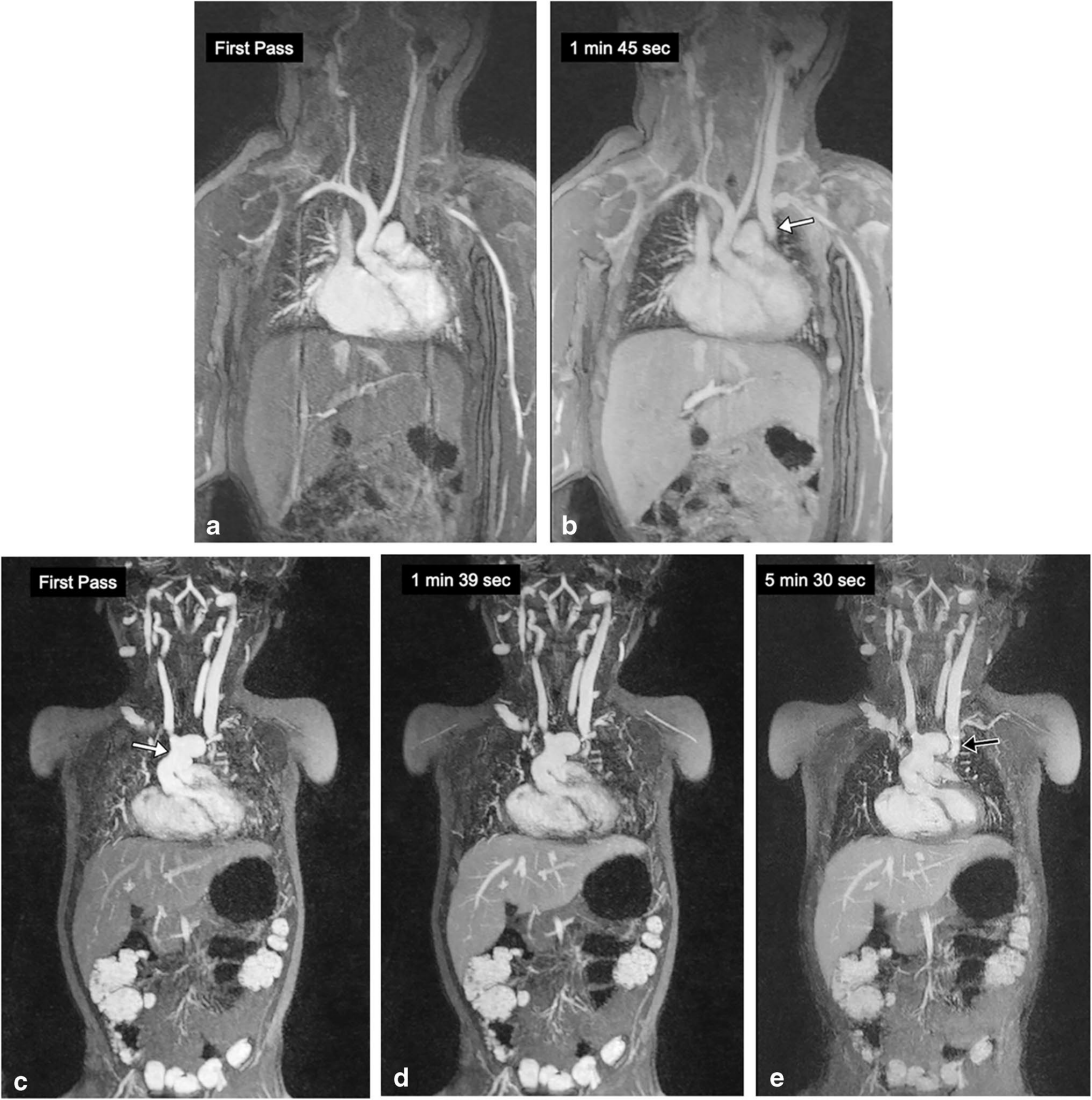
A 3-day-old girl with Shone syndrome. **a, b** Coronal contrast-enhanced magnetic resonance (MR) angiography was performed with gadofosveset at 3.0 T to evaluate vascular anatomy. The arterial phase (**a**) and venous phase (**b**) show good vascular enhancement. Note the left superior vena cava (*arrow* in **b**). **c–e** The same patient was imaged with coronal ferumoxytol-enhanced MR angiography at 3.0 T 2 years after surgery that included a Norwood procedure and bilateral Glenn shunts. The neo-aorta (arrow in **c**) and left Glenn shunt (arrow in **e**) are seen. Note the stable intravascular signal from first pass through 5 min post contrast

Representative GE-MRV and FE-MRV images are provided in Figs. 1, 2, 3, 4, 5, 6, 7, 8 and 9. Figures 1 and 2 show comparable quality in both GE-MRV and FE-MRV in two patients with extensive, systemic venous occlusive disease. Figures 3 and 4 compare GE-MRV and FE-MRV in two patients with portal hypertension and splenomegaly, again showing similar quality in both patients.

Figures 5 and 6 compare two neonatal patients, 2 and 3 days old, respectively. These figures show a noticeable decrease in the intravascular signal with gadofosveset by 2 min, whereas the intravascular signal with ferumoxytol is unchanged after 45 min.

Figure 7 illustrates how coexisting congenital venous anomalies, surgical shunts and veno-occlusive disease can all be assessed on FE-MRV, in this case up to 39 min following ferumoxytol infusion.

Figure 8 shows a 4-year-old dialysis-dependent patient who required venous mapping for catheter access. Repeated cannulation resulted in extensive venous occlusion in the pelvis, chest, neck and bilateral upper extremities. Diseased segments and venous collaterals are clearly visible and uninvolved systemic and portal venous structures in the abdomen and liver are visualized in detail.

### Quantitative assessment

The SNR and CNR for gadofosveset and ferumoxytol are presented in Table 4. SNR and CNR were significantly higher for ferumoxytol than gadofosveset in the IVC (*P* = 0.034 and *P* < 0.001, respectively). The aorta in the venous phase showed significantly higher CNR for ferumoxytol than gadofosveset (*P* = 0.002).

**Table 4:** Quantitative assessment

Quantitative parameter	GE-MRV	FE-MRV	*P*-value
Inferior vena SNR	35.9 ± 11.6	45.1 ± 12.9	0.034
Inferior vena CNR	14.1 ± 7.6	24.6 ± 8.3	< 0.001
Aorta SNR	35.7 ± 13.1	44.1 ± 12.3	0.077
Aorta CNR	13.9 ± 8.6	23.6 ± 8.2	0.002

*CNR* contrast-to-noise ratio, *FE-MRV* ferumoxytol-enhanced magnetic resonance venography, *GE-MRV* gadofosveset-enhanced magnetic resonance venography, *SNR* signal-to-noise ratio

## Discussion

The results of our study show that, compared to gadofosveset, ferumoxytol produces more intense and sustained venous enhancement in the chest, abdomen and pelvis in a pediatric cohort. Once ferumoxytol was distributed throughout the blood pool, the vascular signal did not change noticeably up to the longest time interval sampled (1 h and 13 min). Conversely, the blood pool signal with gadofosveset showed variability over time and among subjects.

The technical success and high diagnostic quality using both agents are reflected in the high SNR, CNR and qualitative scores for image quality and vessel definition. As both groups had similar MRI acquisition parameters and contrast doses, and respiratory motion was well controlled in both groups, the observed qualitative and quantitative comparisons likely reflect true differences between the contrast agents. The venous SNR and CNR of ferumoxytol was significantly higher than gadofosveset, with corresponding higher image quality scores by the two independent radiologists. Although both gadofosveset and ferumoxytol work well as intravascular contrast agents, there are fundamental differences in their kinetics [19]. The intravascular residence time of gadofosveset, as well as its T1 relaxivity, is dependent on the degree of reversible binding to serum albumin. The unbound fraction of gadofosveset is distributed into the extravascular fluid space and is renally eliminated within hours. On the other hand, ferumoxytol remains strictly intravascular, based on its particle size of 30 nm; it does not leak into the extracellular fluid space, it does not interact with serum albumin and it is not excreted by the kidneys. Rather, it is taken up slowly by the reticuloendothelial system and incorporated into the hemopoietic pathway. The variability in vascular signal due to gadofosveset may have been influenced by variability in the degree of protein binding or expansion of the extracellular fluid space due to edema. Patients with low serum albumin would be expected to express lower T1 relaxivity and a more rapid falloff in vascular signal, due to a correspondingly smaller bound fraction of gadofosveset. In our study, we did not correlate signal changes with gadofosveset to serum albumin status, so we regard this hypothesis as speculative rather than evidence based.

The relative dosing with gadofosveset and ferumoxytol warrants mention. Because of its high relaxivity, single-dose gadofosveset, as approved by the FDA and marketed commercially, was 0.03 mmol/kg and this dose was commonly employed for first-pass MR angiography. However, for steady-state blood pool imaging, our center opted for a larger dose of 0.06 mmol/kg. Normalizing for T1 relaxivity, the molar dose of ferumoxytol corresponding to 4 mg/kg is 0.07 mmol/kg [20, 21], which is the dose we employed in our study. Using similar doses based on molar relaxivity and imaging in steady-state eliminates the potentially confounding effects of bolus shape and first pass kinetics, making it easier to compare the blood pool characteristics and temporal signal behavior of the two agents. Differences in vascular signal evolution between the agents should therefore reflect the extravascular redistribution of gadofosveset and its renal elimination.

With regard to overall image quality and motion artifacts (respiratory and pulsation), both gadofosveset and ferumoxytol performed well (average overall image scores > 2). The majority of the younger patients were imaged with controlled ventilation. Controlled apnea is successful in eliminating respiratory motion artifacts with contrast-enhanced MR angiography, resulting in improved image quality and spatial resolution [22], and has been used safely for prolonged breath-holding [23, 24]. Older patients in our study were able to cooperate with breath-hold instructions.

Our results have several important implications. First, we provide evidence that ferumoxytol performed at least as well as gadofosveset, which is significant because ferumoxytol is now the only available MRI blood pool agent in the U.S. Second, ferumoxytol eliminates the need for bolus timing and provides a wider time window for image acquisition, without concerns about signal decay due to redistribution or elimination. This latter unique property can be especially useful in awake pediatric patients where repeated acquisitions may be necessary. As an example, one of the older patients (a 15-year-old) in this study underwent repeated breath-held FE-MRV acquisitions due to motion artifact (images not presented), and the acquisition involving the least motion artifact was selected for review.

An important aspect to consider when deciding on the appropriate choice of contrast media is the safety profile. Nephrogenic systemic fibrosis associated with GBCAs is regarded as an increasingly rare complication, but recent reports have raised concerns about deposition of gadolinium in brain and bone, primarily associated with linear GBCAs [25, 26]. The clinical significance, if any, of this latter phenomenon has not been established, but the potential for unknown long-term effects in children causes unease [27]. Also, early safety reports on ferumoxytol raised concerns for serious hypersensitivity reactions with bolus administration at therapeutic doses [15, 28]. However, a recent multicenter study by Nguyen et al. [29] including 3,215 patients showed no serious adverse events and a 1.9% rate of mild or moderate adverse events at diagnostic doses. This rate of minor adverse reactions is lower than listed in the package insert for the macrocyclic GBCA. Although ferumoxytol was administered as a bolus injection in our study, no adverse events were observed. However, the fact that ferumoxytol (and gadofosveset) were administered in sedated or anesthetized patients may mask symptoms of minor infusion reactions. Nonetheless, objective vital signs and physiological signals were unaltered with both agents, confirming that no serious reactions occurred. Moreover, uniformly high image quality in the veins is present when ferumoxytol is fully distributed in the steady-state, and this phase, once established, is independent of the rate at which the agent was delivered [16, 30]. Another theoretical limitation with ferumoxytol stems from its long time for clearance via the reticuloendothelial system, where T2 signal changes in the liver can persist for weeks [31]. If previous administration of ferumoxytol or other intravenous iron therapy agents is unknown to the radiologist, T2 signal changes in the liver may cause confusion, as described in a case report by McCullough et al. [32]. However, the patient in question had received two therapeutic doses of ferumoxytol (1,200 mg) in a short interval, which even after correcting for weight would be four times the diagnostic dose used in our study. We did not note any similar incidents in our review.

Our study has limitations. Ideally, the cohort of patients evaluated with the two agents would be the same, making direct intra-subject comparison possible. In practice, a fully controlled study such as this is difficult to perform in children and the choice of agent was driven by the clinical questions at the time of use. Only one patient in our cohort was studied with both agents, separated by 20 months (Fig. 9). Nonetheless, both groups were otherwise well matched with regard to age and size. Second, we did not routinely have an independent measure of truth, such as catheter venography, to assess diagnostic accuracy. However, in clinical practice, technically successful MR venography has become the de facto diagnostic standard in children, such that catheter venography is avoided unless for interventional purposes.

## Conclusion

Compared to gadofosveset, ferumoxytol produced higher-quality venous images in children, with more intense and sustained venous enhancement. Ferumoxytol is a practical and effective alternative to gadofosveset, and by extension to the extracellular GBCA, for venographic evaluation in the pediatric age group.

## Supplementary Information

Below is the link to the electronic supplementary material.

Supplementary file1 (DOCX 15 KB)

Supplementary file2 (DOCX 16 KB)
