# Supplementary material for: High-resolution three‑dimensional contrast‑enhanced magnetic resonance venography in children: comparison of gadofosveset trisodium with ferumoxytol
Source: Pediatr Radiol. 2021 Dec 22;52(3):501–12. doi: 10.1007/s00247-021-05225-2 (PMC8857136; doi:10.1007/s00247-021-05225-2)
Supplement: Supplementary file 1 — Supplementary file1 (DOCX 15 KB) [file 247_2021_5225_MOESM1_ESM.docx]

**Online Supplementary Material 1** Vessel definition scoring system

| Overall image quality | 1 = Poor image quality with severe artifact resulting in non-diagnostic images  2 = Moderate image quality with moderate artifact degrading diagnostic content  3 = Good image quality with mild artifact not interfering with diagnostic content  4 = Excellent image quality with no artifacts |
| --- | --- |
| Vessel grading | 1 = Vessel not assessable due to poor image quality  2 = Vessel visualized but only gross features (size/patency) confidently assessable  3 = Vessel well defined and evaluable for structural pathology with high confidence  4 = Excellent vessel definition with sharp borders such that fine details can be evaluated with high confidence |
