# Supplementary material for: High-resolution three‑dimensional contrast‑enhanced magnetic resonance venography in children: comparison of gadofosveset trisodium with ferumoxytol
Source: Pediatr Radiol. 2021 Dec 22;52(3):501–12. doi: 10.1007/s00247-021-05225-2 (PMC8857136; doi:10.1007/s00247-021-05225-2)
Supplement: Supplementary file 2 — Supplementary file2 (DOCX 16 KB) [file 247_2021_5225_MOESM2_ESM.docx]

**Online Supplementary Material 2** Cardiovascular anomalies in gadofosveset and ferumoxytol patient cohorts

| **Gadofosveset** | **Ferumoxytol** |
| --- | --- |
| Patent ductus arteriosus | Patent ductus arteriosus |
| Atrial septal defect | Patent foramen ovale |
| Ventricular septal defect | Ventricular septal defect |
| Truncus arteriosus | Dextro-transposition of the great arteries |
| Total anomalous pulmonary venous return | Tetralogy of Fallot |
| Ebstein anomaly | Hypoplastic left heart syndrome |
| Shone complex | Aberrant right coronary artery |
| Aortopulmonary window | Kawasaki disease with coronary artery aneurysms |
| Hypoplastic left heart syndrome | Large vessel vasculitis with associated dilated cardiomyopathy |
| Hypoplastic aortic arch | Hypoplastic aortic arch |
| Aortic coarctation | Vascular ring (double aortic arch) |
| Vascular ring (right aortic arch with aberrant left subclavian artery) | Left-sided aortic arch |
| Azygos continuation of the inferior vena cava | Left-sided superior vena cava |
